# Edward Tyson, M.D., F.R.S., 1650–1708

**Published:** 1944

**Authors:** 


### EDWARD TYSON, M.D., F.R.S., 1650–1708

*Anatomist and Anthropologist.*

Professor Ashley Montagu, Associate Professor of Anatomy in Hahnemann Medical College and Hospital, has recently published a full study of Dr. Edward Tyson's life and work, in which he has made this great anatomist and anthropologist the central figure in a full and well informed account of the rise of human and comparative anatomy in England.

"Here," says Ashley Montagu, "was a very great man indeed, one whose contribution to human thought and welfare, to science, and to medicine, alone entitled him to rank among the greatest figures of his own or any other day."

Edward Tyson was born in the parish of St. Nicholas, Bristol, on the 20th of January, 1650. His father (also Edward) was a citizen of standing who at various times held the offices of Sheriff, Alderman and Mayor of Bristol; his grandfather (another Edward Tyson) who lived in Bristol and afterwards at Clevedon, was a man of considerable wealth who came of an old Cumberland family. Dr. Edward Tyson was educated in private schools in Somerset, then proceeded to Magdalen Hall, Oxford, where he took the Degrees of B.A., M.A., and Bachelor of Medicine.

The remainder of his life was spent in London, but he did not forget his native city, for he gave £100 to St. Peter's Hospital when it was opened in 1696 by the Corporation of the Poor of Bristol, and he made a gift to St. Stephen's Church, known as "Dr. Tyson's Sermon Gift," settling a sum of money to perpetuate the annual sermon founded "by his grandfather Mr. Tyson of this parish." Later he gave further donations to St. Peter's Hospital.

Edward Tyson was one of the original members of the Royal Society and made many scientific contributions to its meetings, mainly of an anatomical nature. Ashley Montagu holds him high, as one of the first to insist on the gradational conception of the relations of living things. As a dissector and anatomical observer Tyson was unsurpassed, but Ashley Montagu gives him credit for having founded the study of comparative anatomy and the methodical investigation of the natural history of animals. The work which in his own day brought him the widest reputation was the book entitled "Orang-Outang, sive Homo Sylvestris, or the Anatomy of a Pygmie, compared with that of a monkey, an Ape and a Man," published in 1699.

Huxley described this as "the first account of a man-like ape which has any pretensions to scientific accuracy and completeness."

It was based on a dissection of a young Chimpanzee which had been landed in Bristol in a dying state.

But Tyson did not stop at Natural History or comparative anatomy. Before his time purposeful autopsies were unknown. He showed what could be achieved in this way by carefully observing and describing the course and the symptoms of disease and comparing these with the findings upon the autopsied body of one who had succumbed to the disease.

He described anatomical anomalies such as horseshoe kidney and double ureters; he gave in 1683 the first clear picture of the character of the tapeworm and the round worm. He discovered the true nature of hydatids and cysticerci. Curiously enough, the mucilaginous glands of the penis, which he discovered and to which his name is attached, were not the subject of any communication or publication of his own. William Cowper his collaborator in anatomical studies mentioned in passing that Tyson had discovered these glands.

Then, to Ashley Montagu's regret, Tyson became overwhelmed with the claims of a busy London practice and appointment as physician to Bethlehem Hospital. At this Royal Hospital he was one of the greatest reformers and a great benefactor.

The sympathetic treatment of the patients which he introduced is now one of the traditions of the place. Moreover he organized after-care and follow up of discharged cases in order to prevent relapse. In this province of medicine he left an example, but no published writings.

Tyson never married; he was a man of notable taciturnity, "a most understanding anatomist, and a good physician, yet" an Irish acquaintance, Thomas Molyneux, relates "in strange company modest even to an imperfection." But Molyneux found he had some humanity about him, "his studies were his chief delight, only he took now and then a touch at fishing."

As Professor George Sarton of Harvard says in his foreword, "Ashley Montagu has given us in a generous way a full-length portrait of Edward Tyson for which we are grateful."

The enthusiasm and meticulous care with which he has studied every piece of Tyson's work and every contemporary mention of him are indeed admirable.

We are grateful indeed to be reminded of this illustrious son of Bristol.

### REFERENCES.

*Edward Tyson, M.D., F.R.S.*, 1650–1708, *and the rise of human and comparative anatomy in England ; A Study in the History of Science*, by M. F. Ashley Montagu, Assocate Professor of Anatomy, Hahnemann Medical College and Hospital, Philadelphia. *American Philosophical Society Memoirs*, Vol. XX. Price $5.00.

